# The B cell death function of obinutuzumab-HDEL produced in plant (*Nicotiana benthamiana* L.) is equivalent to obinutuzumab produced in CHO cells

**DOI:** 10.1371/journal.pone.0191075

**Published:** 2018-01-11

**Authors:** Jin Won Lee, Woon Heo, Jinu Lee, Narae Jin, Sei Mee Yoon, Ki Youl Park, Eun Yu Kim, Woo Taek Kim, Joo Young Kim

**Affiliations:** 1 Department of Systems Biology, College of Life Science and Biotechnology, Yonsei University, Seoul, Republic of Korea; 2 Department of Pharmacology and Brain Korea 21 Project for Medical Science, Yonsei University College of Medicine, Seoul, Republic of Korea; 3 College of Pharmacy, Yonsei Institute of Pharmaceutical Sciences, Yonsei University, Incheon, Republic of Korea; 4 Department of Integrated OMICS for Biomedical Sciences, Yonsei University, Seoul, Republic of Korea; Chang Gung University, TAIWAN

## Abstract

Plants have attracted attention as bio-drug production platforms because of their economical and safety benefits. The preliminary efficacy of ZMapp, a cocktail of antibodies produced in *N*. *benthamiana* (*Nicotiana benthamiana* L.), suggested plants may serve as a platform for antibody production. However, because the amino acid sequences of the Fab fragment are diverse and differences in post-transcriptional processes between animals and plants remain to be elucidated, it is necessary to confirm functional equivalence of plant-produced antibodies to the original antibody. In this study, Obinutuzumab, a third generation anti-CD20 antibody, was produced in *N*. *benthamiana* leaves (plant-obinutuzumab) and compared to the original antibody produced in glyco-engineered Chinese hamster ovary (CHO) cells (CHO-obinutuzumab). Two forms (with or without an HDEL tag) were generated and antibody yields were compared. The HDEL-tagged form was more highly expressed than the non-HDEL-tagged form which was cleaved in the N-terminus. To determine the equivalence in functions of the Fab region between the two forms, we compared the CD20 binding affinities and direct binding induced cell death of a CD20-positive B cells. Both forms showed similar CD20 binding affinities and direct cell death of B cell. The results suggested that plant-obinutuzumab was equivalent to CHO-obinutuzumab in CD20 binding, cell aggregation, and direct cell death via binding. Therefore, our findings suggest that Obinutuzumab is a promising biosimilar candidate that can be produced efficiently in plants.

## Introduction

A recent breakthrough in cancer treatment employs immunotherapy with monoclonal antibodies that bind to a highly expressed target on cancer cells[1, 2]. In contrast to radiation therapy or chemotherapy, immunotherapy specifically kills cancer cells and has very few side effects[3]. However, the cost of producing monoclonal antibodies for anti-cancer immunotherapy is much higher than for producing small molecule drugs[4]. The protein production system in plants has been demonstrated as a cost efficient and easy-to-scale-up platform for generating bio-drugs[5, 6]. More importantly, in addition to being cost efficient, the use of plants for protein production completely eliminates the risks of virus or residual protein contamination, which are often associated with mammalian production systems[7, 8].

The success of the plant-produced antibody cocktail, ZMapp, in the 2014 Ebola outbreak supported the superiority of the plant protein production system, especially for antibody production[9, 10]. To eliminate plant-specific glycosylation, ZMapp antibodies were produced in transgenic plants by inhibiting expression of xylosyl-transferase and fucosyl-transferase using RNA interference (RNAi)[11]. Unexpectedly, ZMapp antibodies possessed a bulky glycosylated residue (GnGn) in the Fc region, which conferred benefits such as increased FcRγIII affinity and highly enhanced ADCC (antibody-induced cell cytotoxicity)[10, 12]. Very interestingly, a similar improvement in efficacy was found in the development of Obinutuzumab, one of the bio-betters of rituximab[3, 13, 14]. Obinutuzumab is a glyco-engineered antibody generated in Chinese hamster ovary (CHO) cells that overexpress two glycosylation enzymes (β1,4-N-acetylglucosaminyltransferase III and α-mannosidase II)[14, 15]. Obinutuzumab possesses highly bulky glycosylated residues in the Fc region and lacks 1-,6-fucose, which leads to increased FcRγIII affinity and greatly enhanced ADCC activity[3, 16]. Accordingly, elimination of plant-specific oligosaccharides is necessary for antibody production in plants that also can serve as a platform for increasing ADCC activity.

While the N-glycosylation process in animals and plants is relatively well known, differences in post-transcriptional processes between animals and plants have not yet been elucidated[17]. The Fab portion of antibodies have wide sequence variation and may induce a sequence-specific post-transcriptional process. For example, O-glycosylation occurs very differently in animals and plants[5, 7, 17, 18]. Given the fact that biosimilars are basically assessed at equivalent titres, it is important to make sure that the antibodies produced in plants have equivalent performance compared to the original antibodies.

Plant-specific glycosylation of proteins has hampered the rapid industrialisation of plant-produced bio-drugs. Although there has been some argument for the lower allergenicity of proteins produced in plants compared to proteins produced in other organisms[19], there have been no clinical trials to demonstrate the safety of plant-produced monoclonal antibodies in humans; thus, concerns about the immunogenicity of plant-produced monoclonal antibodies in humans remain. Recently, Li et al. reported that the TALEN-mediated gene modifying system used to delete xylosyl transferases and fucosyl transferases in transgenic plants failed to completely delete the xylose and fucose residues in the Fc region of rituximab[20]. At present, the use of RNAi-mediated knockdown to reduce enzyme activities in transgenic plants is the best way to inhibit plant-specific glycosylation. The main bottleneck in pharmaceutical research is plant-specific glycosylation, which will be solved in the near future, but will take time to elucidate. The KDEL/HDEL sequence tag, used localize proteins in the ER, is one method to eliminate plant-specific glycosylation[21, 22]. HDEL-tagged Ig produced in plants was more than 90% high mannose, which was similar to antibodies produced in mammalian systems[23]. ER retention increased the yields of monoclonal antibodies and prevented cleavage of proteins in the apoplasts of *N*. *benthamiana* leaves[24, 25].

Obinutuzumab is currently considered the best treatment for non-Hodgkin’s lymphoma; Obinutuzumab is a type II anti-CD20 antibody that can induces B cell death just via physical binding to target, CD20 [13, 26]. This suggests that Obinutuzumab without Fc mediated immunogenicity can have function for B cell depletion. Thus, although the Fc mediated immunogenicity was not achieved, the antibody removing plant-specific glycosylation by tagging the HDEL, which relieved the possibility of an allergenic response (antibody with HDEL tag has the high mannose type of oligosaccharide which found in mammals), can be capable of causing B cell death. Conversely, if the Fab function of Obinutuzumab produced in *N*. *benthamiana* leaves can be demonstrated as equivalent to that produced in CHO cells, this result would indicate that the protein sequence of the Fab portion of Obinutuzumab is not altered by plant-specific post-transcriptional processes. If this indeed is the case, it would be very easy to produce Obinutuzumab in a variety of transgenic plants or via highly productive expression systems that have solved the issue of plant-specific glycosylation.

## Materials and methods

### Construction of obinutuzumab expression vectors in *N*. *benthamiana* and CHO cells

Light and heavy chain sequences of Obinutuzumab were obtained from GenBank (EA770939) and long chain cDNAs were synthesised by Bioneer (Daejeon, Korea) and plant codon-optimised according to the manufacturer’s protocol (Bioneer, http://eng.bioneer.com, Daejeon, Korea). Original and plant codon-optimised light and heavy chains were inserted into the pCAMBIA 1300 binary vector (p35S:BiP ss-plant-obinutuzumab light chain/heavy chain-HDEL). For ER localisation, the signal sequence of BiP (BiP ss) and the ER retention signal sequence, HDEL, were ligated into the N-terminal and C-terminal regions, respectively. Codon and amino acid sequence are described in supplementary figures (S1 to S3 Figs). The resulting constructs (35S:BiP ss-plant codon-optimized light chain Obinutuzumab, 35S:BiP ss-plant codon-optimized heavy chain Obinutuzumab, 35S:BiP ss- plant codon-optimized heavy chain Obinutuzumab:HDEL, 35S:BiP ss-original light chain Obinutuzumab, 35S:BiP ss-original heavy chain Obinutuzumab, and 35S:BiP ss-original heavy chain Obinutuzumab:HDEL) were transformed into Agrobacterium GV3101 competent cells using the freeze-thaw method into *N*. *benthamiana* (*N*. *benthamiana L*.) plants [27, 28]. PCR followed by sequencing of products was performed to verify the constructs. To produce Obinutuzumab and rituximab in CHO cells (ATCC^®^ CCL-61^™^, ATCC) the light and heavy chains of original Obinutuzumab and rituximab were inserted into the pLenti6.1 vector (ThermoFisher Scientific), then each clone was transfected into HEK cells to generate lentiviruses. HEK cells (ATCC^®^ CRL-3216^™^, ATCC) grown in media containing lentiviruses with light and heavy chain inserts were mixed and used to infect CHO cells. In this way, CHO cells produced light and heavy chain-assembled Obinutuzumab and rituximab.

### Agrobacterium-mediated infiltration into *N*. *benthamiana* leaves

*N*. *benthamiana* plants, aged 4–5 weeks, were used for infiltration. Each light and heavy chain construct was individually transformed into the GV3101 strain of Agrobacterium via the freeze-thaw method. Transformed Agrobacteria were incubated in YEP medium containing 50 mg/ml kanamycin and 50 mg/ml rifampicin at 28°C for 2 days. Agrobacteria were resuspended in infiltration solution (10 mM MES, pH 5.7, 10 mM MgCl_2_, and 500 μM acetone) and infiltrated into the abaxial side of leaves using a syringe as described previously[29]. Infiltrated leaves were harvested after 3–4 days for all experiments.

### Preparation of crude total leaf protein extracts

Wild-type and infiltrated *N*. *benthamiana* leaves were ground with a mortar and pestle under liquid nitrogen to extract crude protein. The powder was suspended in protein extraction buffer containing 50 mM Tris-HCl (pH 7.2), 150 mM NaCl, and protease inhibitor cocktail (Sigma-Aldrich) followed by incubation for 15 min at 4°C[30]. The protein suspensions were centrifuged twice at 13,200 g for 20 min at 4°C and the supernatants were collected. Total protein was quantified using Bradford reagent.

### SDS-PAGE and immunoblot analysis

Total leaf protein extracts (12–20 mg protein) were analysed via SDS-PAGE under reducing conditions in 12% polyacrylamide gels. After SDS-PAGE, the gels were transferred onto PVDF membranes (Merck Millipore, Billerica, MA, USA). The membranes were blocked for 30 min with 5% skim milk in T-TBS buffer containing 50 mM Tris-HCl (pH 7.6), 150 mM NaCl, and 0.01% Tween 20. For immunodetection of expressed Obinutuzumab antibodies, anti-human Ig Fc-specific HRP-conjugated antibody (1:2000 dilution; Abcam) and anti-mouse Ig-specific HRP-conjugated antibody (1:2000 dilution, 109-035-008; Jackson Laboratory) were used. Rubisco recombinant protein standard (Sigma-Aldrich) was used as loading control for Coomassie staining. Protein signals were detected and quantified using chemiluminescence detector LAS-4000 (GE Healthcare, Little Chalfont, UK) and the ImageQuant program (GE Healthcare), respectively. To measure the yield of Obinutuzumab expressed in *N*. *benthamiana* leaf cells, purified CHO K1 cell-expressed rituximab (12.5, 25, 50, 100, and 200 ng) was used as a positive control. The immunoblot signals were quantified using the Bio-Imaging Analyzer (Image Gauge 3.0; Fuji Film, Tokyo, Japan).

### plant-obinutuzumab purification from crude *N*. *benthamiana* leaf protein extracts

Agro-infiltrated *N*. *benthamiana* leaves were harvested and Obinutuzumab was purified as follows. Total soluble proteins were extracted with two volumes of plant protein extraction buffer (50 mM Tris-HCl pH 7.2, 150 mM NaCl), and a protease inhibitor cocktail (Sigma-Aldrich); the extract was centrifuged at 16,000 g for 20 min. The supernatant was filtered through two layers of Miracloth to remove insoluble material then re-centrifuged at 16,000 g for an additional 30 min. The clarified extract was filtered through 0.2 μm pore filters (Sartorius Stedim Biotech GMBH, Gottingen, Germany) then loaded onto a protein A affinity chromatography column (Pierce, GE Healthcare Life Sciences, Baie d’Urfe, Quebec, Canada). The column was washed with extraction buffer, and antibodies were eluted using 100 mM glycine pH 3.0. Elution fractions were immediately neutralised with 2 M Tris-HCl (pH 7.4) and analysed using Ponceau S staining and western blot to determine antibody quantity and purity. Antibodies were stored at -80°C until used.

### Confocal fluorescence microscopy analysis

Wild-type and Obinutuzumab-infiltrated *N*. *benthamiana* leaves were fixed for 3 days with 3.7% paraformaldehyde in PBS (pH 7.2). Some samples were co-infiltrated with a p35S:BiP ss-RFP (red fluorescence protein)-HDEL construct to indicate ER localisation. The fixed leaves were paraffin-embedded, sectioned at 10 μm thickness, and adhered to gelatine-coated slides. The anti-human Ig Fc-specific FITC-conjugated antibody (109-095-008, Jackson Laboratories, 1:2000 dilution in blocking solution: 5% horse serum, 5% goat serum, 1% gelatine in PBS) was applied to the samples at room temperature for 30 minutes, and the slides were washed three times with PBS. Nuclei were stained with DraQ (ab108410, Abcam). FITC, RFP, and DraQ fluorescence was evaluated using confocal microscopy (Zeiss 780, Jena, Germany).

### Binding affinity test using flow cytometry analysis

To evaluate binding specificity, mCherry-CD20-expressing HEK cells were stained with the various purified antibodies. Anti-human Ig Fc-specific FITC-conjugated antibody was used as a secondary antibody and DAPI was used for nuclear staining. FITC, mCherry, and DAPI fluorescence were imaged by confocal microscopy (Zeiss 780). To compare the binding affinities of the antibodies, flow cytometry (FACS, BD Biosciences, Franklin Lakes, NJ, USA) was used. Ramos cells (5 x 10^5^) were stained with plant-obinutuzumab-HDEL or CHO-obinutuzumab at 1, 10, 100, 1000, and 10,000 nM for 15 minutes followed by anti-human Ig Fc-specific FITC-conjugated secondary antibody (109-095-008, 1:200 dilution; Jackson Laboratories) for 30 minutes at 4°C. Cells were twice washed with culture medium then analysed. An anti-human Ig Fc-specific FITC-conjugated antibody only-treated sample was used for the negative control. The binding was measured as the mean fluorescence intensity of each sample.

### Analysis of CD20 aggregation and lipid raft co-localisation in Ramos cells

Ramos lymphoma cells were grown in RPMI with 10% FBS, and 1% penicillin/streptomycin. Cells (2 x 10^5^) in RPMI were incubated with 10 μg/ml of each antibody for 30 min at 37°C in 5% CO_2_. After washing, cells were incubated with anti-human Ig Fc-specific FITC-conjugated antibody (Jackson-109-095-008, 1:200 dilutions in blocking solution). Cells were fixed with 3.7% paraformaldehyde in PBS for 10 min. To visualise the localization of each antibody in lipid rafts, goat anti-caveolin 1 antibody (EnoGene, E18-6386-2, 1:100) and anti-goat Ig-specific Alexa 568-conjugated secondary antibody (Invitrogen, A-11057, 1:200) were used. FITC and Alexa 568 fluorescence were observed using confocal microscopy (Zeiss 780).

### Ramos cell depletion via direct antibody binding

Ramos cells were resuspended in RPMI media and plated on a round-bottom 96-well plate (at a density of 5 x 10^5^ cells/well). The respective antibody dilution was added and incubated for 24 hours at 37°C and 5% CO_2_. To stain live cells, calcein AM (Invitrogen, C34852) was freshly dissolved in DMSO (final concentration 10 nM) and added to each well. The retention of calcein in live cells was used as a readout by flow cytometry (FACS).

### Statistical analyses

Data are presented as the means ± standard error of the mean. Statistical analysis was performed by Student’s t-test followed by Tukey’s multiple comparisons using the GraphPad Prism software package (version 5.0), as appropriate. P<0.05 was considered statistically significant.

## Results

### Obinutuzumab binary vector construction for plant-based monoclonal antibody expression

**Fig 1:**
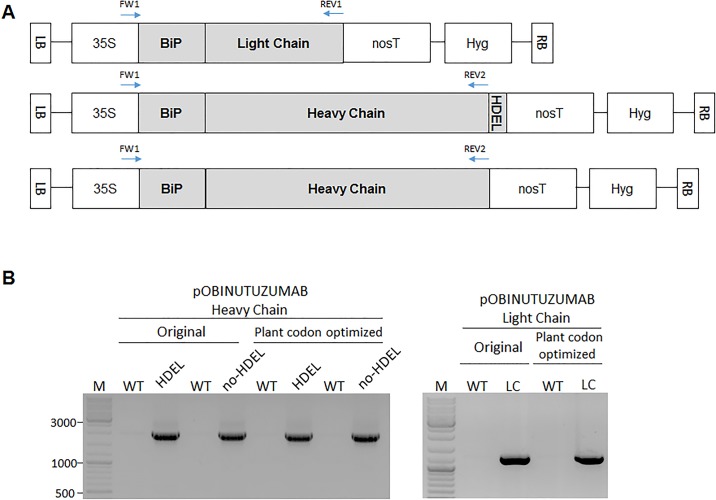
Light and heavy chains obinutuzumab constructed with or without an HDEL tag were successfully incorporated into the *N*. *benthamiana* genome. (A) Schematic representation of plant-obinutuzumab Heavy Chain and Light Chain to produce in *N*. *benthamiana* leaves by using transient infiltration method. Each construct (BiP ss-light chain, BiP ss-heavy chain-HDEL, and BiP ss-heavy chain) was used 35S, cauliflower mosaic virus 35S promoter; BiP ss, ER targeting signal sequence; HDEL, ER retention signal; NOS, nos terminator; Hyg, hygromycin-resistance cassette; LB, left border; RB, right border. Binding regions of each set of primers used in Fig. 1A are indicated by pairs of red arrows. (B) Validation of constructs incorporated into the *N*. *benthamiana* genome. Genomic DNA was extracted from *N*. *benthamiana* leaves engineered to express each construct via agrobacterium-mediated infiltration and was used as template for PCR with primer pairs indicated in Fig. 1A (S4 Fig for primer sequences). (PCR product sizes: light chain, 1350 bp; heavy chain, 2050 bp).

To express Obinutuzumab in *N*. *benthamiana* leaves, we obtained protein sequences from a drug bank (https://www.drugbank.ca) and patent information (US20050123546). We synthesised the nucleotide sequences of the full-length light and heavy chains as original mammalian codon and plant codon optimised sequences (S2 and S3 Figs, S1 Fig for amino acid sequence with signal sequences). In order for the heavy and light chains to assemble in the ER, the localisation signal sequence from the ER protein BiP was attached to the N-terminal region of the light and heavy chains. Meanwhile, the ER retention signal sequence HDEL was attached to each chain to permit accumulation in the ER (Fig 1A). To confirm the incorporation of each construct into the *N*. *benthamiana* genome via agrobacterium-mediated infiltration, the vector inserts were verified by PCR. The genomic DNA of each construct infiltrated into *N*. *benthamiana* leaves was used as template with the primer pairs indicated in Fig 1A (arrows, S4 Fig for sequences). The proper sizes of PCR products confirmed that the heavy and light chain constructs were stably incorporated into the *N*. *benthamiana* genome via agrobacterium-mediated infiltration (Fig 1B).

### Comparison of the integrity, expression level, and cellular localisation of plant-obinutuzumab-HDEL and plant-obinutuzumab in *N*. *benthamiana* leaves

**Fig 2:**
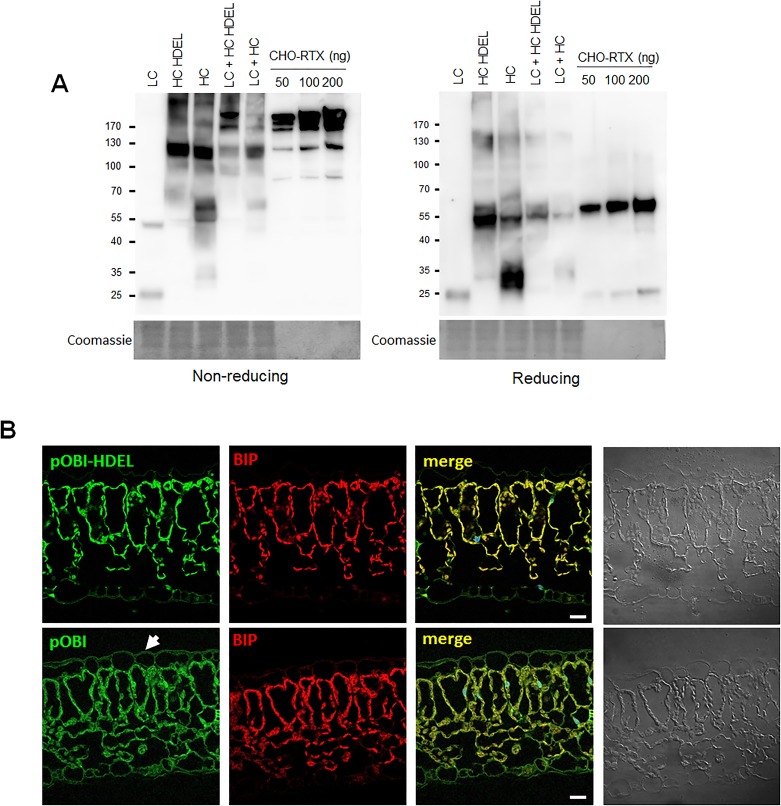
Comparison of the integrity and expression of light and heavy chains with or without HDEL. (**A**) Total protein extracts were subjected to PAGE under non-reducing and reducing conditions. Immunoblot was performed using HRP-conjugated human Ig Fc-specific antibody and HRP-conjugated mouse Ig Fc-specific antibody to detect both chains. CHO-rituximab (50 ng, 100 ng, and 200 ng) was used as the standard. Coomassie-stained gel images were used to show equivalent loading of proteins. (**B**) Comparison of localization and expression between plant-obinutuzumab-HDEL and plant-obinutuzumab from *N*. *benthamiana* leaves. Immunohistochemistry was performed to detect the localisation of plant-obinutuzumab-HDEL and plant-obinutuzumab in *N*. *benthamiana* leaves. Formalin-fixed and paraffinised *N*. *benthamiana* leaves expressing plant-obinutuzumab-HDEL and plant-obinutuzumab were sectioned at 10 μm thickness and immunostained. Fluorescein isothiocyanate (FITC; green)-conjugated anti-human Ig Fc-specific 2^nd^ antibody was used for detection. BiP protein fused with Ds-RED (red) was used to indicate the localisation of ER in *N*. *benthamiana* leaves. DraQ was used to indicate the nucleus (blue). Bar: 20 μm.

Total protein extracts were separated by polyacrylamide gel electrophoresis (PAGE) under reducing and non-reducing conditions followed by immunoblot using an HRP-conjugated anti-human Ig Fc specific antibody, and an HRP-conjugated mouse Ig-specific antibody. To verify the expression of each construct, the amount of total protein in each extract (8 μg) was compared to 50 ng and 100 ng purified CHO cell-produced rituximab (CHO-RTX), as control (Fig 2A). After comparing the protein expression level of original and plant codon-optimized constructs (S5 Fig), we used the original sequence heavy chain and light chain constructs for further experiments, because those showed slightly increased expression level than plant codon optimized constructs. In case of the no-HDEL heavy chain, a ~35 KD truncated protein was detected, which implied that the heavy chain secreted into apoplasts was cleaved by an unknown proteinase, as previously reported[25, 31]. On the other hand, the heavy chains with HDEL-tags did not show any truncated forms. These data suggested that the light and heavy chain-HDEL-infiltrated *N*. *benthamiana* leaves successfully expressed plant-obinutuzumab-HDEL with similar biochemical protein properties to Rituximab. To confirm the localization of each antibody in *N*. *benthamiana* leaves, plant-obinutuzumab-HDEL and plant-obinutuzumab were evaluated using immunohistochemistry with FITC-conjugated human Ig-specific antibodies. To indicate the location of the ER, RFP (red fluorescent protein)-tagged BIP was co-infiltrated with plant-obinutuzumab-HDEL or plant-obinutuzumab. Fig 2C shows that most plant-obinutuzumab-HDEL and plant-obinutuzumab were found in the ER, but plant-obinutuzumab-HDEL showed more protein expression in the ER than plant-obinutuzumab.

### The yield of plant-obinutuzumab-HDEL produced in *N*. *benthamiana* leaves

**Fig 3:**
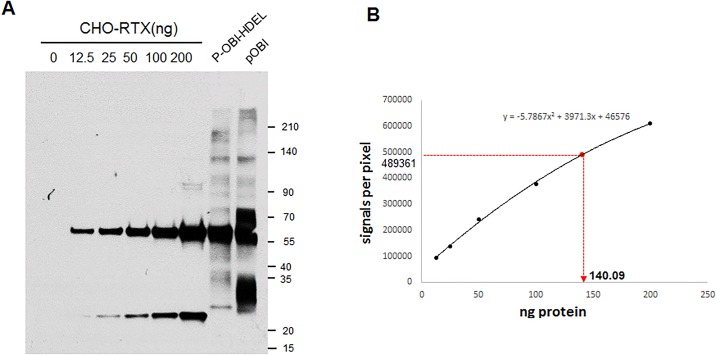
Quantification of plant-obinutuzumab-HDEL production yield in *N*. *benthamiana* leaves. (A) Various amounts (1, 12.5, 25, 50, 75, 100, and 200 ng) of CHO-obinutuzumab and each 20 μg of total soluble protein extract from the leaves expressed plant-obinutuzumab-HDEL and plant-obinutuzumab-no HDEL were subjected to immunoblot with human and mouse IgG specific-HRP conjugated antibody. The heavy chain bands of CHO-obinutuzumab were quantified to obtain a standard curve then expression amounts of each antibody was estimated by the heavy chain intensities of total soluble lysate. (B). The immunoblot band of 20 μg plant-obinutuzumab-HDEL of total soluble protein extracted in *N*. *benthamiana* leaves was used to calculate the production yield.

To measure yields, various amounts of CHO-RTX were loaded with 20 μg total soluble protein from *N*. *benthamiana* leaves expressing HDEL-tagged and untagged antibodies, and immunoblot analysis using HRP-conjugated human Fc-specific antibodies was performed. According to the standard curve derived from rituximab, the production yields of plant-obinutuzumab-HDEL comprised approximately 140 ng out of 20 μg of total soluble proteins, which corresponded to ~0.7% of total proteins (Fig 3). In the case of plant-obinutuzumab, the heavy chain appeared to have been cleaved in the Fab region because the truncated heavy chain was co-purified, which implied the Fc portion remained intact to bind protein A. We did not calculate the production yield of plant-obinutuzumab and we used plant-obinutuzumab-HDEL for further experiments.

### Specific epitope recognition of plant-obinutuzumab-HDEL

**Fig 4:**
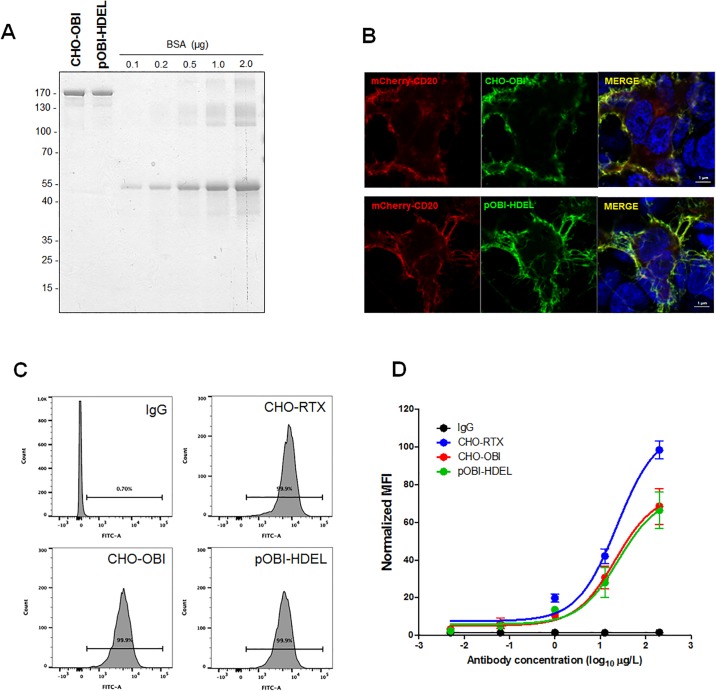
Specificity and affinity of epitope binding by plant-obinutuzumab-HDEL compared to CHO-obinutuzumab. (**A**) Antibody protein concentrations were measured via BCA method and quantification of PAGE gel analysis with Coomassie blue staining. To validate the concentration of each antibody, 1 μg of antibodies was subjected to PAGE under non-reducing conditions with BSA (0.1, 0.2, 0.5, 1, 2 μg) as the standard. Gels were then stained with Coomassie blue. (**B**) Specific epitope recognition by plant-obinutuzumab-HDEL was tested by immunocytochemistry. mCherry-tagged CD20 was expressed in HEK cells. CHO-obinutuzumab and plant-obinutuzumab-HDEL were used for immunocytochemistry with FITC-conjugated human Fc-specific secondary antibodies. Bar 1 μm. C. Representative FACS images for affinity comparison with 10 μg/ml antibodies. (**C, D**) Dose-dependent binding capacity of CHO-rituximab, plant-obinutuzumab-HDEL and CHO-obinutuzumab (1 ng/ml, 10 ng/ml, 100 ng/ml, 1 μg/ml, and 10 μg/ml) using flow cytometry. Representative FACS images of binding affinity with 10 μg/ml antibodies are shown in **C**. FITC intensities of 10 μg/ml of each antibody bound to cells are depicted by normalized mean fluorescence intensity (MFI). Results of triplicate assays are summarised in **D.**

To confirm the specific epitope recognition of plant-obinutuzumab-HDEL, immunocytochemistry analysis was performed with CHO-obinutuzumab as a control. The mCherry-CD20 plasmid was transfected into HEK cells and immunocytochemistry was performed with plant-obinutuzumab-HDEL and FITC-conjugated secondary human Fc-specific antibodies. plant-obinutuzumab-HDEL only bound to mCherry CD20-expressing HEK cells, similar to CHO-obinutuzumab. In addition, flow cytometry analysis was performed to compare their binding affinities (Fig 4C shows representative images). Incubation of Ramos cells (CD20-positive B cell line) with 10 μg/ml plant-obinutuzumab-HDEL or CHO-obinutuzumab resulted in similar binding affinities. A dose-dependent increase in affinity is shown and summarised in Fig 4D. CHO-obinutuzumab and plant-obinutuzumab-HDEL showed similar binding affinities, which were about half of the affinity of rituximab-CHO; IgG showed no binding at any dose. These data clearly indicated that plant-obinutuzumab specifically bound to CD20 and the binding affinity was equivalent with to CHO-obinutuzumab.

### plant-obinutuzumab and CHO-obinutuzumab binding induced similar CD20 and B cell changes

**Fig 5:**
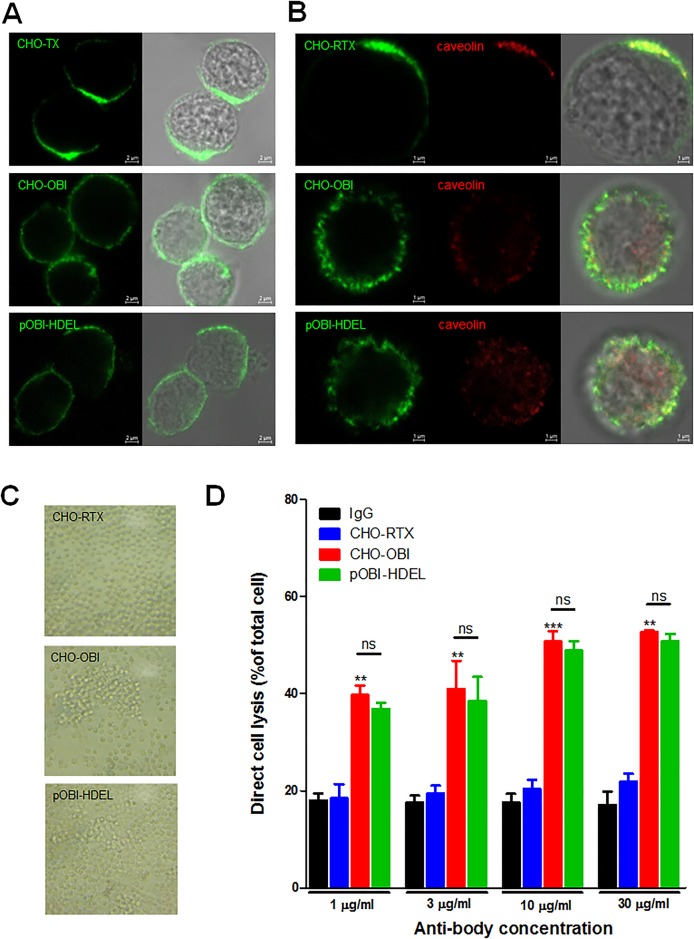
plant-obinutuzumab-HDEL binding to Ramos cells showed typical type II CD20 antibody characteristics, similar to CHO-obinutuzumab. (**A**) Two-panel photomicrograph showing binding of the antibodies to CD20 localized on the surface of Ramos cells. Binding was visualised with FITC-conjugated human Fc-specific secondary antibody. Bar, 2 μm. (**B**) In addition to the same antibodies used in A, caveolin was stained with anti-caveolin antibody and Alexa 568-conjugated secondary antibody. Merged DIC image shows CD20 and caveolin co-localised on the surface of the Ramos cell. Bar, 1 μm. (**C**) Photomicrograph showing cell aggregation (homotypic adhesion: HA) 30 minutes after treatment with each antibody. (**D**) Direct binding-induced cell death caused by CHO-obinutuzumab and plant-obinutuzumab-HDEL were compared to IgG and CHO-rituximab. Each antibody (at 1 μg/ml, 10 μg/ml, and 30 μg/ml) was incubated with Ramos cells for 14 hours and cell death was measured by lose of calcein-AM dye via FACS analysis. Three independent experiments are shown as means ± s.e.m. **P < 0.01; ***P < 0.001.

The binding of rituximab to CD20 induces polarisation of lipid rafts and tight clustering of caveolins; however, Obinutuzumab binding to CD20 does not induce polarisation of lipid rafts[3, 14]. To determine whether plant-obinutuzumab-HDEL had a similar effect, co-localisation of CD20 and caveolin was evaluated (Fig 5). Ramos cells were incubated with 10 μg/ml plant-obinutuzumab-HDEL or CHO-obinutuzumab for 15 minutes, fixed with a 4% paraformaldehyde PBS solution, and immunostained with a FITC-conjugated human Fc-specific secondary antibody. As shown in Fig 5, after treatment with plant-obinutuzumab-HDEL or CHO-obinutuzumab, CD20 was distributed in a punctate manner on the cell surface, while CD20 was distributed in clusters on the surface of Ramos cells incubated with rituximab-CHO (Fig 5A). In addition, caveolin aggregation also was found with CD20 on rituximab-CHO treated cells, but not CHO-obinutuzumab and plant-obinutuzumab-treated cells (Fig 5B).

Obinutuzumab causes homotypic adhesion (HA) of cells and DBCC-mediated B cell death via binding to CD20[3, 13, 14]. As shown in Fig 5C, plant-obinutuzumab and CHO-obinutuzumab resulted in HA that was not observed with rituximab-CHO. Most interestingly, the rate of cell death induced by direct binding of plant-obinutuzumab and CHO-obinutuzumab was very similar; cell death was not observed in rituximab-CHO-treated or IgG control-treated cells (Fig 5D). These data clearly demonstrated that plant-obinutuzumab-HDEL and CHO-obinutuzumab had similar effects on B cells via binding to CD20. The similarities in CD20 binding activities (Fig 4) and the effect on Ramos cells (Fig 5) demonstrate that plant-obinutuzumab-HDEL and CHO-obinutuzumab have equivalent epitope binding ability, and implies no plant specific modification in their Fab regions.

## Discussion

Obinutuzumab is a monoclonal antibody used to deplete CD20-expressing lymphoma cells and B cells via ADCC and direct binding[3, 13–15]. Of the 16 different anti-CD20 antibodies clinically available at present[32], Obinutuzumab is recognized as superior based on low minimal residual disease (MRD) and increased progression-free survival[2]. Therefore, the demand for Obinutuzumab is expected to be high. The anticipated demand for Obinutuzumab will be a significant opportunity to demonstrate the superiority of plant antibody production platforms. Our binding affinity test showed that plant-obinutuzumab-HDEL and CHO-obinutuzumab showed similar binding to CD20 on Ramos cells, and furthermore, both antibodies showed similar extend of the B cell depleting activity via physical binding to CD20 that are typical characteristics of type II anti-CD20 antibodies[32]. Accordingly, we suggest that plant-obinutuzumab-HDEL can be used to kill CD20-expressing lymphoma cells just by direct binding without complements or effector cells, as similar to CHO-obinutuzumab.

In our study, plant-obinutuzumab-HDEL was expressed at a ratio of 0.7% of the total soluble protein, and was purified at a ratio of ~55%. Previous studies have shown that the yield of IgG in *Nicotiana benthamiana* ranges from 105.1 μg/g (IgG for HIV virus[33]) to 500 μg/g (IgG for Ebola virus GP1)[34] depending on the expressed protein. Compared to previous reports, the average yield of plant-obinutuzumab-HDEL, 14.8 μg/g, was relatively low. To increase yields, a special high yield multi-protein expression system is required, such as the BeYDV (bean yellow dwarf virus)-derived DNA replicon system[34].

Our data agreed with previous reports that the HDEL tag in IgG promotes accumulation of the monoclonal antibodies in the ER (Fig 3), resulting in high expression[21, 23, 35], but also protects the antibodies from possible cleavage within apoplasts. The HDEL tag resulted in high mannose-type glycosylation, which accounted for more than 80% of total N-glycans; Man_7_GlcNAc_2_, the same type of glycosylated residue found in humans[23], was most abundant. A small amount of xylose residue (~5%), acquired during transport from non-ER compartments, was also present in HDEL-tagged IgG[23]. However, small amounts of xylose have low probability to cause any immunogenic responses in humans. In support, Elelyso, a plant-produced recombinant glucocerebrosidase, has not been reported to cause any dangerous events[19]. On the other hand, plant-obinutuzumab not tagged with HDEL showed truncation of the protein in its N-terminal region, which resulted in unusable Fab-deleted antibodies (Fig 3A). There are reports that the IgG secreted within apoplasts undergoes cleavage through specific aspartic, cysteine, and serine peptidases, and the acidic pH in the extracellular space facilitates these proteolytic activities in *N*. *benthamiana* [25, 31]. If the pH of the total protein extract during purification is maintained at a high enough level, it is possible to solve the antibody cleavage issue.

The immunogenicity and hypersensitivity caused by plant-specific glycosylation are still highly debated[19], but no evaluation of bio-drugs can be too strict for human safety. In addition, plant-specific glycosylation is considered the main obstacle for rapid industrialisation of plant-produced bio-drugs. Until now, there have been no reports of human studies that show plant-derived proteins are more immunogenic than mammalian-derived bio-therapeutics[19]. Nevertheless, careful observation of the progress toward the use of plant-derived proteins is needed for more efficient and rational development of plant-produced bio-drugs. Li et al. reported that TALEN-mediated knock-out of xylosyl-transferase and fucosyl-transferase was unsuccessful in eliminating all xylose and fucose residues in Obinutuzumab due to compensatory activity likely from other isotypes of xylose and fucose transferases[20]. While Cas-9-mediated genome editing shows the most promise for eliminating these enzymes, it requires a significant amount of time to perform. At present, the use of RNAi in transgenic plants to inhibit expression[11] of xylosyl-transferase and fucosyl-transferase is the best way to achieve prevent plant-specific glycosylation. Considering the growth of plants, transgenic plants with all genes deleted by Cas-9 may not be a better production platform than transgenic plants using RNAi. However, a more economical and mammalian protein risk-free production platform is in high demand for the entire bio-drug industry. To this end, a plant-based protein production platform is the most promising approach that ultimately will contribute to worldwide welfare.

There have not been many successful examples of producing antibodies in plants so far. Considering its value, *E*. *coli* is the cheapest protein production host. However, plants are considered to be a better production platform for bio-drugs because of the presence of clean and fresh images of plants as well as post-translational modifications such as glycosylation. Although there are many similarities between the post-transcriptional processes of animals and plants studied so far, differences between the post-transcriptional processes as well as plant-specific O-glycosylation are not completely understood, which creates other possible bottlenecks to production and medical use of plant proteins. Furthermore, there are few reports of equivalence between plant-produced and mammalian proteins, so additional studies are necessary to demonstrate equivalence in order to truly industrialize the use of plants as a medical protein production platform. Our results clearly demonstrated that the Fab portion of plant-obinutuzumab-HDEL had very similar functional characteristics to CHO-obinutuzumab (Fig 5); furthermore, plant-obinutuzumab-HDEL was equivalent to CHO-obinutuzumab with respect to cell death of B cells induced by Fab binding to CD20. Plant-specific glycosylation-free plants can be used to produce Obinutuzumab, which may be an improved bio-better production platform for monoclonal antibodies with strong ADCC capability that are similar to Obinutuzumab produced in glyco-engineered CHO cells.

In conclusion, we demonstrated that Obinutuzumab is a promising candidate as a plant-produced monoclonal antibody by showing that the Fab region of plant-obinutuzumab-HDEL has equivalent ability to bind CD20 and causes direct binding mediated B cell death compared to CHO-obinutuzumab.

## Supporting information

S1 Fig Amino acid sequences of light and heavy chains of CHO-obinutuzumab and plant-obinutuzumabs. (TIF)

S2 Fig Original DNA sequences of light and heavy chain plant-obinutuzumabs. (TIF)

S3 Fig Plant codon optimized DNA sequences of light and heavy chain plant-obinutuzumabs. (TIF)

S4 Fig Primer sequences used for genomic integration of plant–obinutuzumab light and heavy chain constructs. (TIF)

S5 Fig Heavy chain and light chain protein expression by original codon and plant-optimized codon. (TIF)

## Abbreviations

ADCC: antibody-dependent cell-mediated cytotoxicity
BiP: luminal binding protein
CaMV: cauliflower mosaic virus
CDR: complementarity determining region
ER: endoplasmic reticulum
HRP: horseradish peroxidase
