# Correction: The B cell death function of obinutuzumab-HDEL produced in plant (*Nicotiana benthamiana* L.) is equivalent to obinutuzumab produced in CHO cells

**DOI:** 10.1371/journal.pone.0195917

**Published:** 2018-04-11

**Authors:** Jin Won Lee, Woon Heo, Jinu Lee, Narae Jin, Sei Mee Yoon, Ki Youl Park, Eun Yu Kim, Woo Taek Kim, Joo Young Kim

Fig 2, Fig 4, and Fig 5 are incorrect. The authors have provided the corrected versions here.

**Fig 2:**
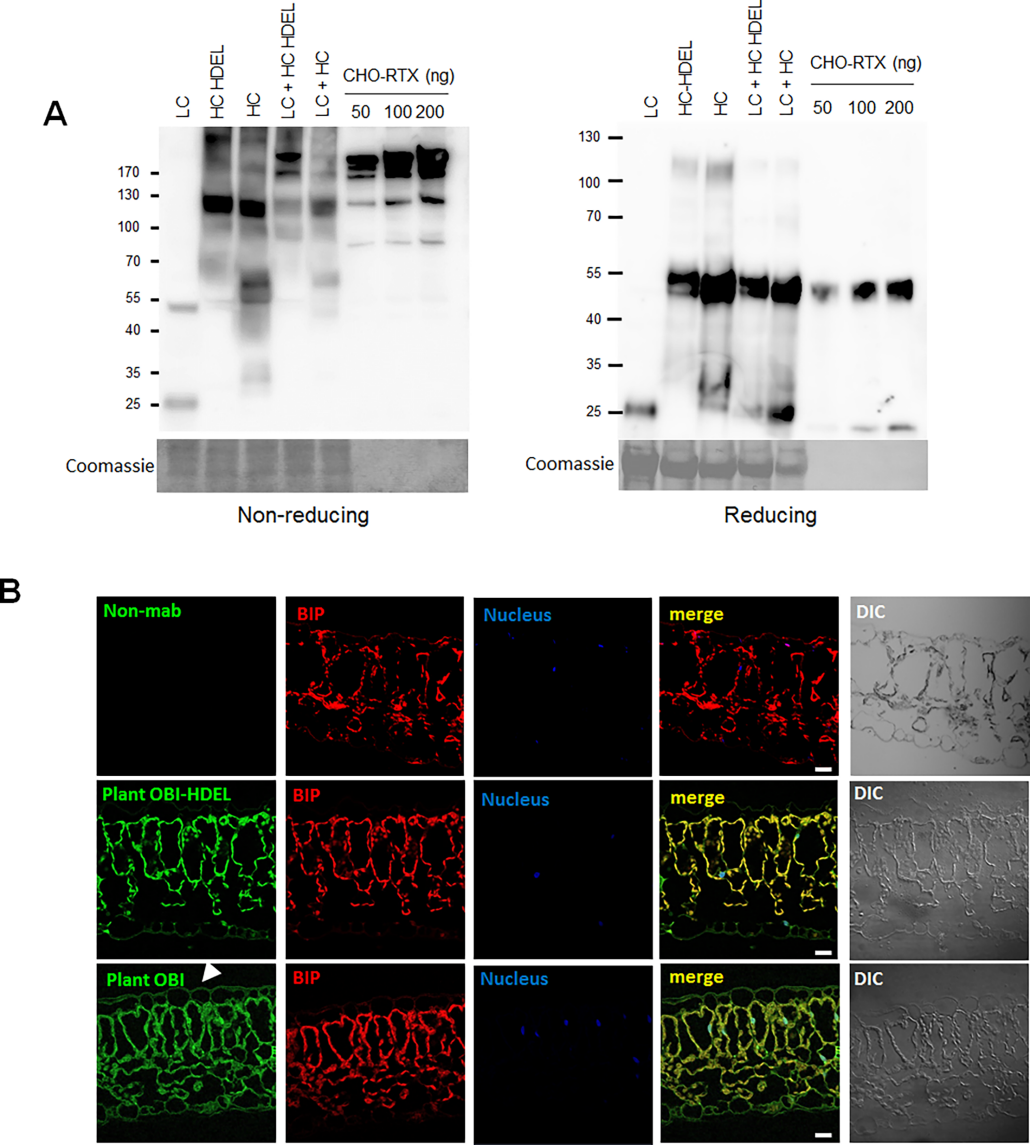
Comparison of the integrity and expression of light and heavy chains with or without HDEL. (A) Total protein extracts were subjected to PAGE under non-reducing and reducing conditions. Immunoblot was performed using HRP-conjugated human Ig Fc-specific antibody and HRP-conjugated mouse Ig Fc-specific antibody to detect both chains. CHO-rituximab (50 ng, 100 ng, and 200 ng) was used as the standard. Coomassie-stained gel images were used to show equivalent loading of proteins. (B) Comparison of localization and expression between plant-obinutuzumab-HDEL and plant-obinutuzumab from N. benthamiana leaves. Immunohistochemistry was performed to detect the localisation of plant-obinutuzumab-HDEL and plant-obinutuzumab in N. benthamiana leaves. Formalin-fixed and paraffinised N. benthamiana leaves expressing plant-obinutuzumab-HDEL and plant-obinutuzumab were sectioned at 10 μm thickness and immunostained. Fluorescein isothiocyanate (FITC; green)-conjugated anti-human Ig Fc-specific 2nd antibody was used for detection. BiP protein fused with Ds-RED (red) was used to indicate the localisation of ER in N. benthamiana leaves. DraQ was used to indicate the nucleus (blue). Bar: 20 μm.

**Fig 4:**
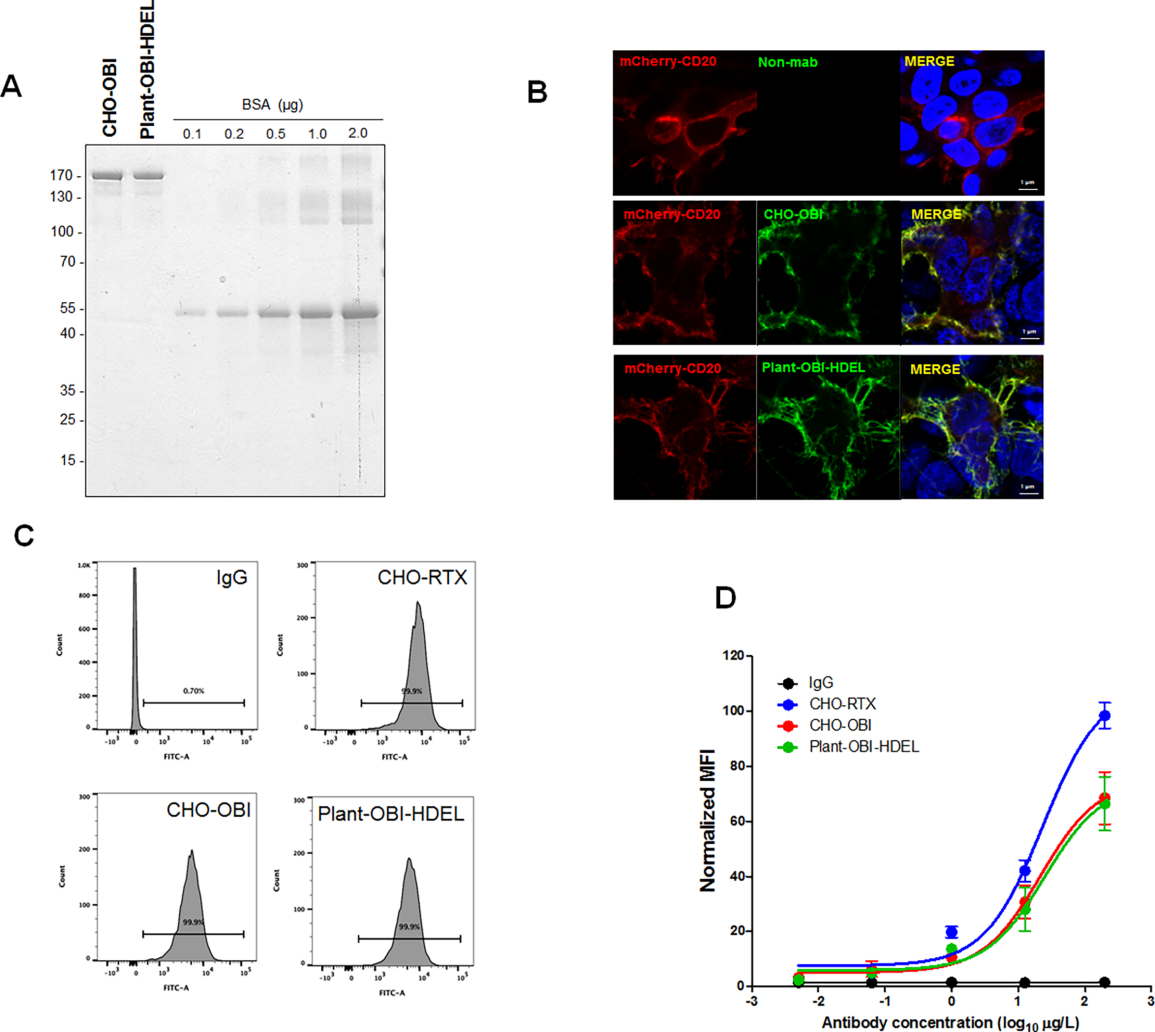
Specificity and affinity of epitope binding by plant-obinutuzumab-HDEL compared to CHO-obinutuzumab. (A) Antibody protein concentrations were measured via BCA method and quantification of PAGE gel analysis with Coomassie blue staining. To validate the concentration of each antibody, 1 μg of antibodies was subjected to PAGE under non-reducing conditions with BSA (0.1, 0.2, 0.5, 1, 2 μg) as the standard. Gels were then stained with Coomassie blue. (B) Specific epitope recognition by plant-obinutuzumab-HDEL was tested by immunocytochemistry. mCherry-tagged CD20 was expressed in HEK cells. CHO-obinutuzumab and plant-obinutuzumab-HDEL were used for immunocytochemistry with FITC-conjugated human Fc-specific secondary antibodies. Bar 1 μm. C. Representative FACS images for affinity comparison with 10 μg/ml antibodies. (C, D) Dose-dependent binding capacity of CHO-rituximab, plant-obinutuzumab-HDEL and CHO-obinutuzumab (1 ng/ml, 10 ng/ml, 100 ng/ml, 1 μg/ml, and 10 μg/ml) using flow cytometry. Representative FACS images of binding affinity with 10 μg/ml antibodies are shown in C. FITC intensities of 10 μg/ml of each antibody bound to cells are depicted by normalized mean fluorescence intensity (MFI). Results of triplicate assays are summarised in D.

**Fig 5:**
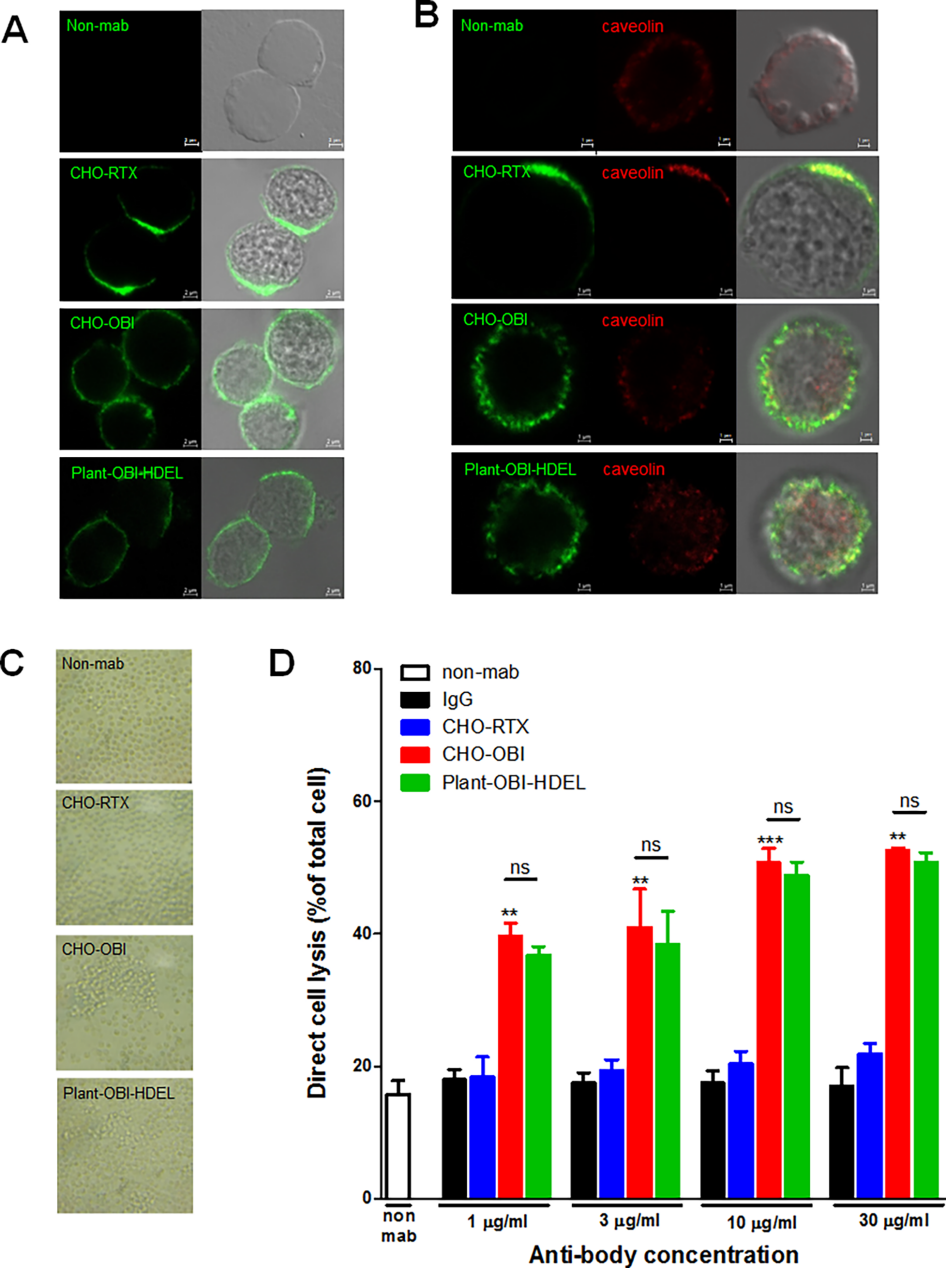
plant-obinutuzumab-HDEL binding to Ramos cells showed typical type II CD20 antibody characteristics, similar to CHO-obinutuzumab. (A) Two-panel photomicrograph showing binding of the antibodies to CD20 localized on the surface of Ramos cells. Binding was visualised with FITC-conjugated human Fc-specific secondary antibody. Bar, 2 μm. (B) In addition to the same antibodies used in A, caveolin was stained with anti-caveolin antibody and Alexa 568-conjugated secondary antibody. Merged DIC image shows CD20 and caveolin co-localised on the surface of the Ramos cell. Bar, 1 μm. (C) Photomicrograph showing cell aggregation (homotypic adhesion: HA) 30 minutes after treatment with each antibody. (D) Direct binding-induced cell death caused by CHO-obinutuzumab and plant-obinutuzumab-HDEL were compared to IgG and CHO-rituximab. Each antibody (at 1 μg/ml, 10 μg/ml, and 30 μg/ml) was incubated with Ramos cells for 14 hours and cell death was measured by lose of calcein-AM dye via FACS analysis. Three independent experiments are shown as means ± s.e.m. **P < 0.01; ***P < 0.001.
