# Supplementary figures and images for: The B cell death function of obinutuzumab-HDEL produced in plant (Nicotiana benthamiana L.) is equivalent to obinutuzumab produced in CHO cells
Source: PLoS One. 2018 Jan 11;13(1):e0191075. doi: 10.1371/journal.pone.0191075 (PMC5764350; doi:10.1371/journal.pone.0191075)

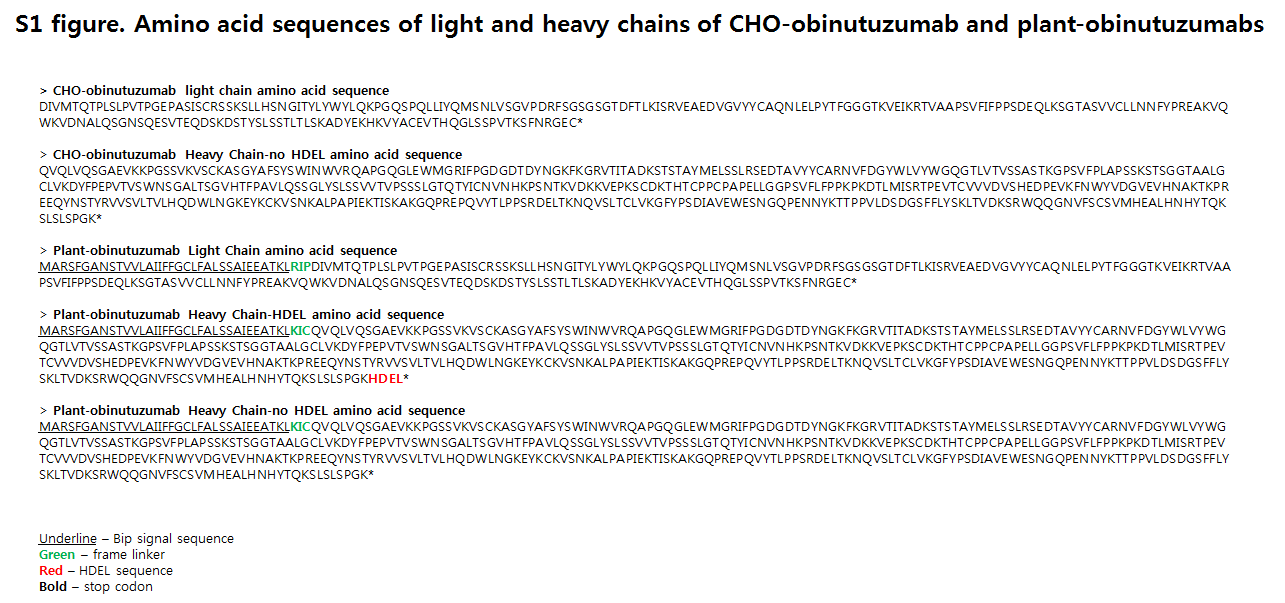

Supplement: S1 Fig — (TIF) [file pone.0191075.s001.tif]

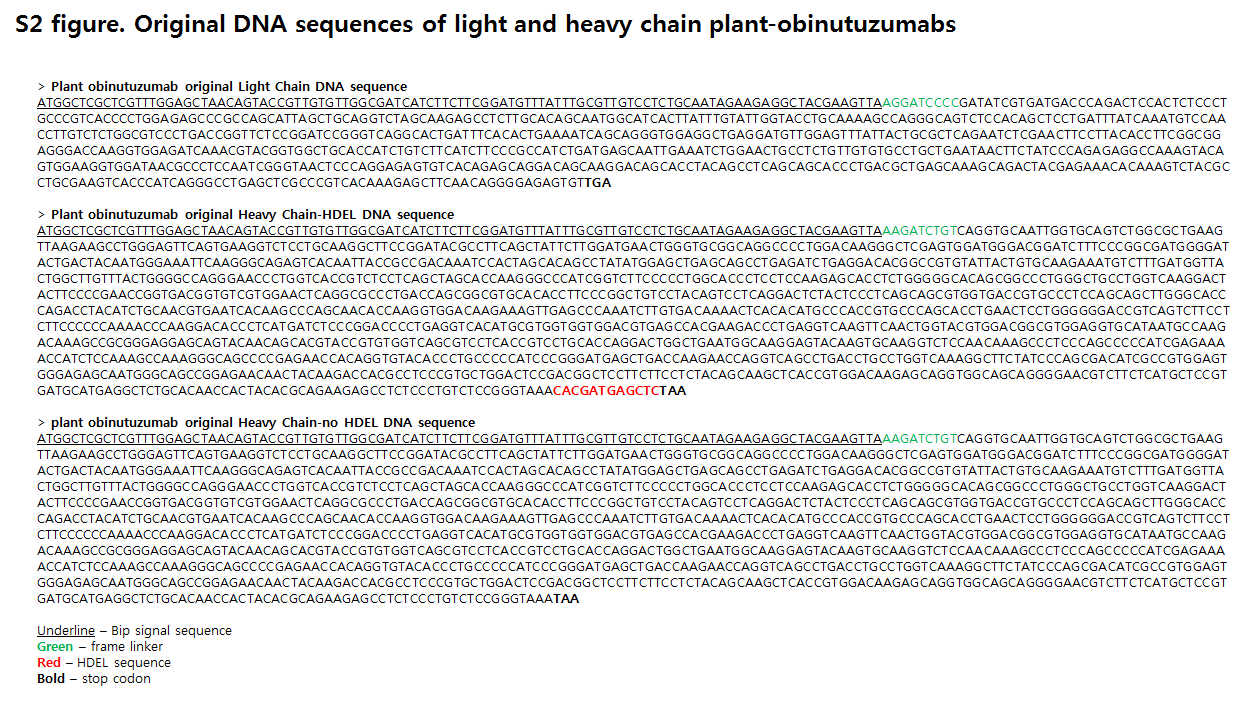

Supplement: S2 Fig — (TIF) [file pone.0191075.s002.tif]

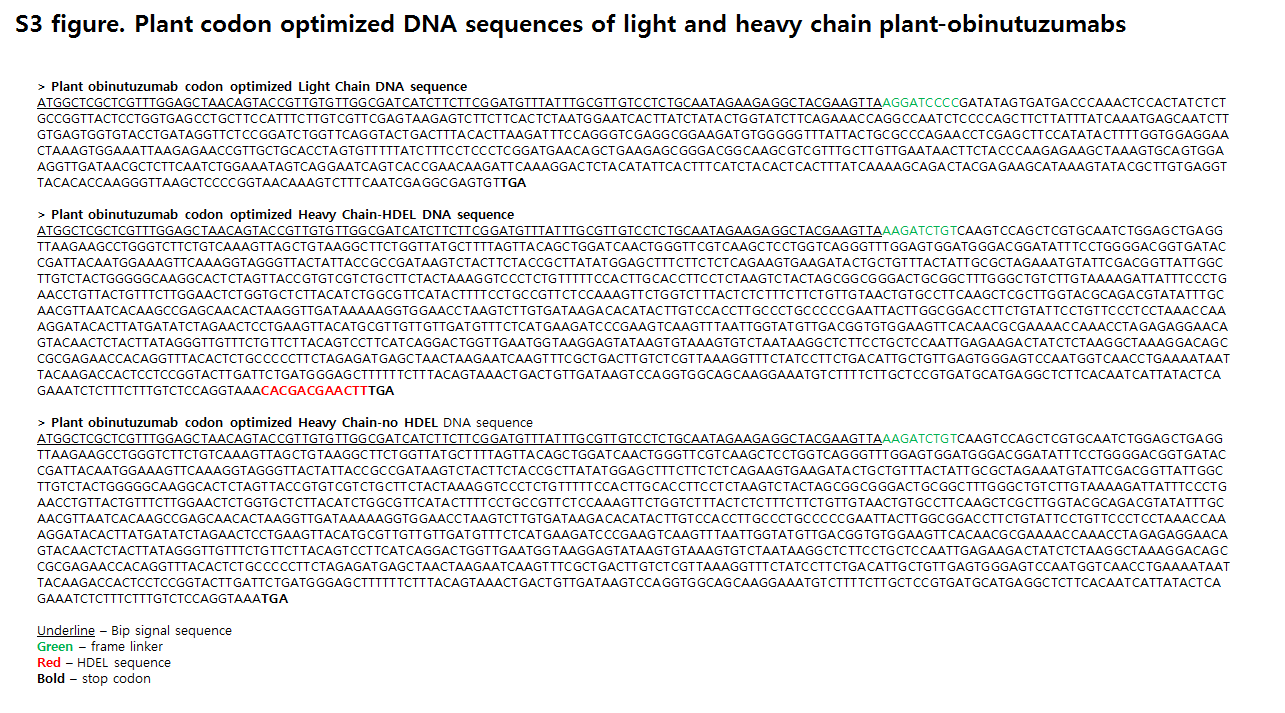

Supplement: S3 Fig — (TIF) [file pone.0191075.s003.tif]

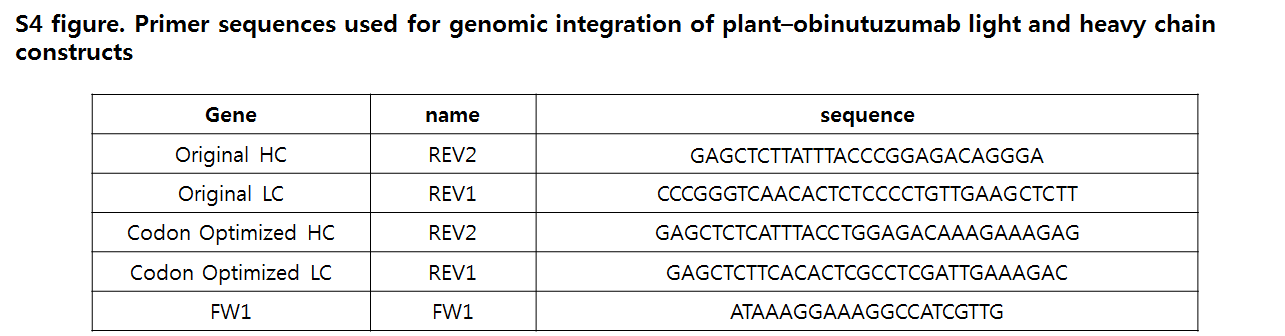

Supplement: S4 Fig — (TIF) [file pone.0191075.s004.tif]

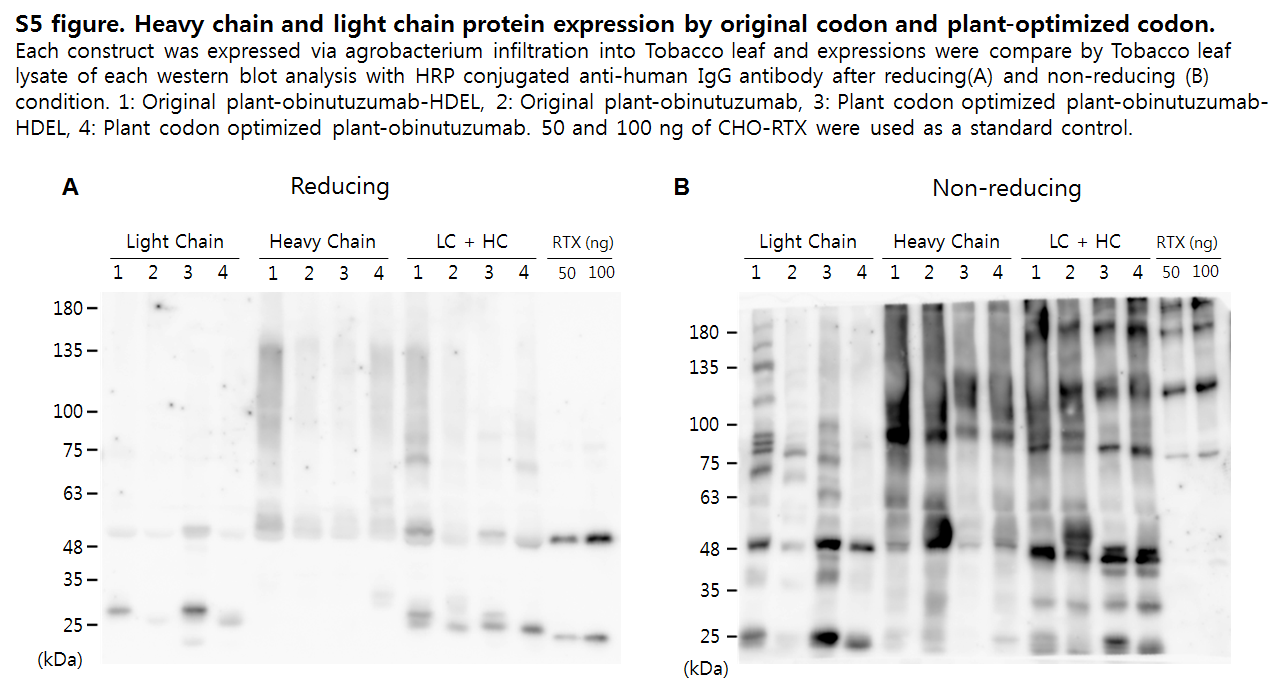

Supplement: S5 Fig — (TIF) [file pone.0191075.s005.tif]
